# Mechanochemical Degradation of Biopolymers

**DOI:** 10.3390/molecules28248031

**Published:** 2023-12-10

**Authors:** László Jicsinszky, Fabio Bucciol, Salah Chaji, Giancarlo Cravotto

**Affiliations:** Department of Drug Science and Technology, University of Turin, 10125 Turin, Italy; fabio.bucciol@unito.it (F.B.); salah.chaji@unito.it (S.C.)

**Keywords:** ball mill, ultrasound, cavitation, extrusion, carbohydrates, polyhydroxylated phenols, lignin, protein

## Abstract

Mechanochemical treatment of various organic molecules is an emerging technology of green processes in biofuel, fine chemicals, or food production. Many biopolymers are involved in isolating, derivating, or modifying molecules of natural origin. Mechanochemistry provides a powerful tool to achieve these goals, but the unintentional modification of biopolymers by mechanochemical manipulation is not always obvious or even detectable. Although modeling molecular changes caused by mechanical stresses in cavitation and grinding processes is feasible in small model compounds, simulation of extrusion processes primarily relies on phenomenological approaches that allow only tool- and material-specific conclusions. The development of analytical and computational techniques allows for the inline and real-time control of parameters in various mechanochemical processes. Using artificial intelligence to analyze process parameters and product characteristics can significantly improve production optimization. We aim to review the processes and consequences of possible chemical, physicochemical, and structural changes.

## 1. Introduction

The term ‘mechanochemistry’ refers to the simultaneous combination of mechanical and chemical transformations that result in changes at the molecular level. The complex processes involve several physical, physicochemical, and chemical processes that can cause an altered reaction mechanism. There are several ways to perform a mechanochemical reaction, such as cavitation, shearing, friction, and grinding [1,2,3,4,5,6]. In many cases, the catalytic processes, known as mechanocatalysis, form a distinct class in mechanochemistry. However, these processes do not fundamentally constitute a separate class since the chemical structure of the catalyst in mechanocatalysis can temporarily change, like in classic catalytic reactions.

The ability to control the response of polymers at the molecular level to macroscopic effects has provided the basis for the emergence of reactive materials. The transformation of natural polymers of cellulosic origin in response to physical impacts, such as nitrocellulose molecular weight reduction, is the consequence of the shortening of the cellulose fibers. Staudinger reported similar observations when grinding polystyrene, cellulose, or nitrocellulose in a colloidal ball mill. The partial de-nitration of nitrocellulose during prolonged grinding suggested the chance of breakage of covalent bonds or new bond formation by purely mechanical forces [7]. Another finding in polymer mechanotransformation is that rubber mastication is not a simple physical disaggregation but an oxidation process [7,8,9,10].

Encina et al. [11] demonstrated the differences between mechanochemically and mechanically induced physical transformations. Later, Ghanem et al. emphasized the power of the atomistic approach in exploiting the potential of reactive polymers [12]. They also found a direct correlation between the number of bond scissions and the viscosities of treated and untreated polymers. Table 1 summarizes the dissociation energies of bonds commonly found in biomolecules. In a polymer backbone, the formed O-O bonds are weaker than C-C bonds, making them more likely to break (ΔE_O-Odissociation_ ≈ 150 kJ/mol (≈36 kcal/mol) vs. ΔE_C-Cdissociation_ ≈ 376 kJ/mol (≈90 kcal/mol)) [13]. Although the studied polymers show loose connections to biopolymers only, the bond strengths provide information on the behavior of biomolecules under mechanochemical conditions. For instance, in the paper industry, the processing of technical pulp strongly alters the fiber length, and reprocessed fibers can become so damaged that the pulp cannot be suitable for paper production anymore. However, the cellulose molecules do not change significantly in the processing, as the dead-ground cellulose has the same degree of polymerization as short-ground or unground celluloses [7].

Simple polymers, such as polyethylene, polystyrene, or polymethacrylate, undergo a homolytic fragmentation of the C-C bond in their backbone. In recent decades, polymer mechanochemistry has developed rapidly, and various scientists have put much effort into creating polymers that elicit more complex and valuable responses or exhibit specific mechanochemical reactions such as mechanochromism and strain deformation. Mechanochromism is a color change phenomenon of solids under mechanical stress, such as grinding, crushing, rubbing, or high pressure and ultrasonication in a solution or dispersed state [17,18]. Similar studies on mechanochemically induced chemical and physicochemical changes are spreading intensively but are still rare, probably due to the great diversity of biopolymers.

All mechanochemical reactions of solid particles contain at least one cracking step. Though various solids may behave differently in grinding, Zhurkov concluded a general equation studying different types of solids, like alloys, non-metallic crystals, and polymers [19]. The lifetime of 50 solids studied under uniaxial tension has suggested a universal rate relation between lifetime, stress, and temperature in the following equation:

τ = τ_0_ × e^(U_0_ − γσ)/kT^, (1)

where τ_0_ is the reciprocal of the natural oscillation frequency of atoms in the solid, U_0_ is the binding energy on the atomic scale, γ is proportional to the disorientation of the molecular structure, σ is the tensile stress, k is the Boltzmann constant, and T is the absolute temperature. Theoretically, all the variable parameters, except the empirical γ, can be calculated by quantum chemical tools, and for polymers, they are derived from electron paramagnetic resonance. The values of τ_0_, γ, and U_0_—the latter shows a linear correlation with σ—are considered constant parameters, and τ_0_ falls in the 10–13 s range, as calculated from experimental data. Thermal motion, which plays a principal role, determines the fracturing of solids. In a body experiencing stress, chemical bonds can break due to thermal fluctuations. The likelihood of this process occurring depends heavily on the magnitude of the tensile stress. This stress promotes the breaking of bonds and decreases the activation barrier. The value of the product γ × σ determines the reduced activation energy barrier [19,20,21].

The Basedow [22] and Zhurkov [19] approaches use mechanochemical kinetic models. However, they only provide a phenomenological description of material systems, as interactions occur at the atomic, molecular, and supramolecular levels. These models define rate constants as a function of mechanical and chemical effects due to their significant contribution to the rate constants. Although the extension of the kinetic theory of strength involves an energy analysis of the deformation and fracture processes of solids and their compounds, it retains the phenomenological description, as shown in Equation (2), as defined by Butyagin and Streletskii [23]. The Q_rel_ term refers to the released energy in the products of the components ground together.

Q_rel_ ≈ (1 − ΔH × G × S) × D_max_, (2)

where G is reaction energy yield per unit area, S is the contact area, ΔH is the reaction enthalpy, and D_max_ is the maximum collision energy.

**Figure 1:**
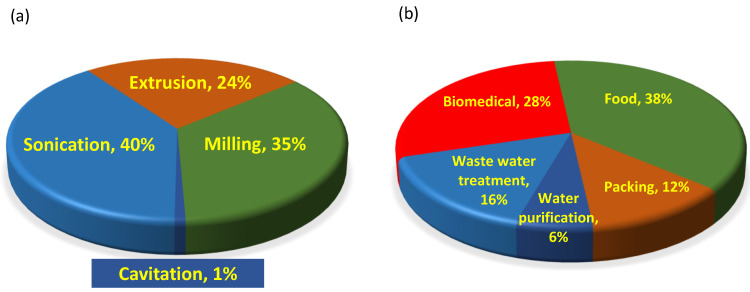
(**a**) Publication distribution of the principal mechanochemical technologies; (**b**) distribution of publications on various application fields of biomolecule mechanochemical transformation.

**Figure 2:**
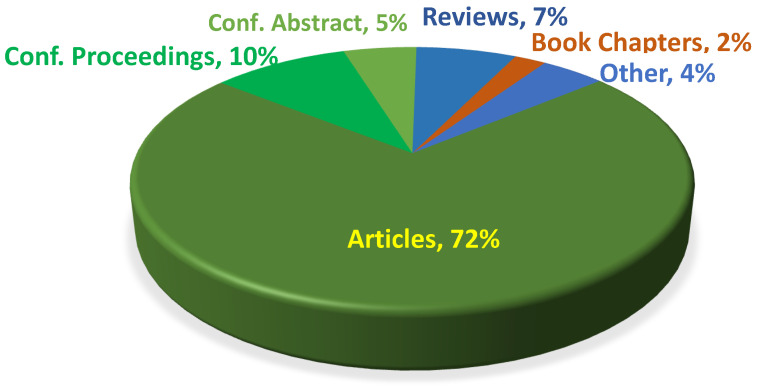
Averaged distribution of scientific papers on mechanochemistry, from searches of various combinatorial keywords: (mill/cavitation/extrusion/(sonication/ultrasonic)/ultrasound)/(mechanochemistry/mechanochemical)/(macromolecule/biomacromolecule) (last: 15 October 2023).

Mechanochemical technologies, including milling, cavitation, and extrusion, can significantly affect the structure of biomolecules. Biopolymer degradation studies show a balanced publication frequency among the most common mechanochemical methods (see Figure 1a). Within the cavitation phenomena, the literature typically distinguishes between hydrodynamic cavitation and sonication-induced cavitation (sonocavitation). These subtypes have many similarities, and the main processes are also the same, but the technologies operated are different. Although hydrodynamic cavitation is known more for its destructive properties [24,25], and the instrumentation is much simpler than sonocavitation, the number of publications on the application of hydrodynamic cavitation to biomolecules is minimal, as shown in Figure 2a. Ultrasonic treatment in many laboratories is used for dissolution acceleration or degassing of HPLC eluents. These processes are physical rather than chemical. Both physical and chemical transformations usually require at least one liquid phase, which limits their application. Sonication has higher application potential than hydrodynamic cavitation as it is less sensitive to the particle size of solids in the liquid phase. Usually, it is hard to make distinctions between chemical reactions on the enlarged solid surface and the better solubilities. The bath and horn technologies are widespread in sonochemistry. Nowadays, the simplest bath version belongs to routine laboratory accessories, while the horn version often requires more complex instrumentation. Unlike the bath version, using an ultrasonic horn probe is usually more efficient in transferring a high-frequency mechanical wave to a solution [26,27,28,29]. The efficacy of the reaction depends on the nature of the liquid medium and often can cause surprising side reactions. The destructive properties of cavitation dominate scientific publications, as shown in Figure 1b. Although extrusion can use liquid—either as a molten phase—or pure solid phases, the food industry typically relies on this technology [30,31,32,33]. Only the fast development of analytical methods can highlight the chemical transformation during extrusion. Nowadays, all of the mechanochemical technologies mentioned above also aim at biofuel production, which combines mechanical and chemical conversion to provide a greener and renewable alternative to fossil energy [34,35,36].

Mechanical stress on polymer molecules may re-entangle the chains, alter the orientation of the chains, or break primary and secondary bonds. Biopolymers can undergo reversible or irreversible transformations that can change their physical as well as their chemical, sensory, and even physiological properties, unlike organic and inorganic polymers building up defined repeating units. This manuscript briefly discusses the primary factors that can impact degradation kinetics in fluids, such as cavitation effects induced by flow or ultrasound. Furthermore, we also discuss the physical and chemical transformations that may arise during grinding and extrusion processes. We review the possible changes based on the most common types of biomolecules and mechanochemical technologies. Not only are the macromolecules produced from biological species but also the chemical transformation of biopolymers or polymers produced from biological resources often considered biopolymers [37,38]. Polymers of bio-based repeating units (lactic acid, caprolactam, polybutylene succinate, etc.) or their materials modified in post-production can be better considered semi-biopolymers and are principally outside the scope of this manuscript.

## 2. Historical Background

Before getting into the technical details, some of the most fundamental events in mechanochemical technology over the past nearly three hundred years are summarized below. Mechanochemistry is not a fundamentally new technique [39]. Although the first report appeared on a mechanochemical organic transformation almost 130 years ago [40], the reaction of biomolecules to mechanical stresses or mechanochemically triggered active species does not have such a long history. 

Three decades after publishing Bernoulli’s law, Euler formalized the current version of Bernoulli’s equation [41]. At the end of the XVIII century, after Venturi published his instrument, [42], Euler first speculated about cavitation (“negative pressure”). The expression cavitation appeared only at the dawn of the XX century, when Thornycroft and Barnaby, as navy ship factory engineers, noticed the intensive bubbling around the propellers [43]. Three decades later, in 1927, Richards recognized that sound waves can induce unusual chemical effects in a liquid, later known as sonochemistry [44]. Wood and Loomis reported in detail the biological and physical consequences of supersonic waves and the devices used to generate them in the same year [44,45]. Despite the early results, the study of sonochemistry remained extremely modest until the 1980s [22,46]. The advent of inexpensive and reliable generators of high-intensity ultrasound (sound having frequencies above human hearing, greater than 20 kHz) resulted in explosion-like developments in sonochemistry. 

The earliest known record of mortar used for mechanochemical purposes dates back to the ancient Greek era, as documented by Theophrastus [47]. Prehistoric discoveries imply a potentially large-scale mercury production, albeit through rudimentary methods by modern standards. Throughout history, grinding devices primarily served for grain milling. However, traditional butter production can exhibit specific mechanochemical characteristics [47,48]. Staudinger was the first to publish a scientific description of the molecular weight reduction in polystyrene subjected to high-speed ball milling or turbulent flow [8]. In the years since, significant advances in polymer synthesis, analytical techniques, and the advent of computerization have transformed polymer mechanochemistry into a science in its own right.

Bramah first patented [49], in 1795, the extrusion process for bulk pipe production from soft metals. The method forced a preheated metal through a die via a hand-driven plunger. The effective lead pipe production method led to the invention of the hydraulic press by Burr and Bramah. After almost a hundred years of a relatively silent period, the bulk production of alloys and foods began to use various kinds of extrusions [50]. The attention turned slowly to the chemical processes during the extrusion even later when the analysis allowed us to study changes both in line (i.e., in various sections of the instrument) and in real time [51,52].

Since the beginning of the documentation of chemical publications, there have been about 6–700,000 technical papers on mechanochemistry and 3–5 times more, with nearly 2,500,000–3,200,000 patents by the major databases (last accessed 15 October 2023), although the number of patents is more uncertain than the number of articles. The distribution of various scientific publications is shown in Figure 2.

The study of the degradation of biomolecules by any mechanochemical treatment has only come to the fore in recent decades. The number of publications on biomolecular transformation mechanisms and kinetics is minimal, nearly 0.5% of all publications, compared to those on common plastics and inorganic materials, despite the extensive studies on their degradation. It is also true that polymers of well-defined subunits, e.g., caprolactam or lactic acid esters and semi-biopolymers, can also provide information on the potential degradation of biomolecules. De Smitt attempted to generalize their degradation studies on polylactic acid semi-biopolymers to polymers containing protonated or esterified carboxylic acid moieties [53]. They assumed that typical reactions may involve both (trans)acylation and degradation transformations in radical reactions. The ultrasonic treatment and γ-irradiation mentioned above show similarities, although the energy sources are very different. Ester bonds can have two potential cleavage points at the CO-O and C-CO bonds. Recombination of radicals can increase the branching character of macromolecules. Although amide bonds, which are more common in proteins, show many similarities to ester bonds, the general transfer of the model to more stable amide bonds is not palpable in publications.

## 3. Theoretical Background and Instrumentation

The difference between electromagnetic irradiation and mechanochemical stress is that the former involves the interaction of electromagnetic waves with matter, while the latter involves mechanical force to induce chemical reactions, as described by the Planck–Einstein relation. The waves carry energy, momentum, and angular momentum, which exert forces on charged particles. Electromagnetic irradiation can also cause changes in the electronic structure and valence state of atoms and molecules. In a recent book, Al-Assaf exhaustively summarized the current knowledge on electromagnetic irradiation-induced chemical and physicochemical transformations of natural polymers based on carbohydrates [54]. Systematic studies of electromagnetic irradiation-triggered acoustic effects have a short history. Laser-beam-generated ultrasound is suitable for various analytical investigations in material sciences. The metal industry uses this technique for measurement and detection of, e.g., grain size, defects, anisotropy, and mechanical properties [55,56], but biomedical applications are in the very early stages [57]. In the early 2000s, the discovery of microwave-induced ultrasonic waves initiated the utilization of thermoacoustic ultrasound generation [58]. Although the utilization of this effect in chemical reactions [59,60,61] and medicinal imaging technology started soon [62], the analysis of the thermo-acoustic effect on biomaterials is sporadic [63,64].

Mechanochemical stresses can induce chemical reactions by lowering the activation energy or increasing the reaction rate. Chemical reactions can be caused by transferring sufficient energy through various methods, such as grinding, milling, shearing, or compressing solids. Mechanochemical stress can cause changes in the crystal structure, phase composition, surface area, and defect concentration of materials or generate reactive species that can interact with the stressed material.

The strength of the force applied from outside determines how much damage is applied to the bond when it is under stress. When dealing with non-covalent mechanochemistry, a few hundred piconewtons (pN) can cause changes in the molecular structure due to the coordinated movement of a specific set of bonds. There is a clear line between non-covalent and covalent mechanochemistry based on the bond nature and force required to break them. However, many biologically relevant processes involved in mechanosensory and mechanotransduction often exhibit force-activated scenarios [65]. Since polymer covalent bond rotation requires hundreds of pN of stress, protein–metal interactions typically require less intensive force to dissociate. This difference highlights the damage of metallated proteins during various types of biomaterial processing in the food industry. On the other hand, mechanical impacts can break crucial hydrogen bonds that provide mechanical stability to biopolymers, such as nucleic acids (<100 pN) and proteins (50–500 pN). In polysaccharides, the monosaccharide units are generally in the chair conformation. Experiments using periodate oxidation have shown that the chair-to-boat conformation of the pyranose ring units can significantly change the elasticity of the polysaccharide chain. The segment elasticity of α(1→4) D-glucopyranoside homopolymers (amyloses, pectins) changes from nearly 6000 pN to about 34,000 pN, and in the case of α(1→6) homopolymers (dextrans), the elasticity changes from about 15,000 pN to nearly 34,000 pN. When the polymer contains both α(1→4) and α(1→6) segments (e.g., pullulan or amylopectin), the elasticity changes from nearly 10,000 pN to about 50,000 pN. The force–extension curves showed hysteresis, demonstrating a reversible conformational change. A similar conformational change in the pyranose ring in β(1→4)-linked polymers (celluloses) is blocked under a stretching force, giving the polysaccharide significantly lower longitudinal elastic properties. The experimental values were in excellent agreement with the quantum chemical calculations [66].

Although the medical applications of ultrasound technology differ significantly from materials science methods, the shear and cavitation effects can also affect biomolecules in living cells, as biomolecules tend to contain less-stable chemical bonds than plastics. For example, ultrasound can modify and control the molecular weight of polymers. The degradation of polystyrene is associated with a shear mechanism, where solvent molecules move around collapsing cavitation bubbles [67].

In covalent mechanochemistry, the covalent bond scission, which includes both homolytic and heterolytic rupturing, typically requires much higher forces, ranging from 0.2 to 10 nN [68]. Understanding the principles of mechanochemical signal transduction will also have implications for many technological fields, from biotechnology to tissue engineering and drug development [69].

The radicals produced in mechanochemical processes are involved in many chemical transformations. Although radicals can exhibit a characteristic optical spectrum (UV, visible, IR) due to their low concentration, the photon absorption of non-radicals can easily mask their signals. Electron spin resonance (ESR) spectroscopy and spin trapping are suitable tools for detecting unpaired electrons at broken bonds, the presence of free radicals, unpaired electrons, or trapped electrons or paramagnetic ions. ESR is highly sensitive because it is not affected by molecules in the matrix, and the detection of radicals is possible if a system contains more than 10^15 free radicals. Radicals exhibit a unique ESR spectrum, allowing for qualitative and semi-quantitative identification. Spectral intensities also provide information on the radical concentration, but since it is difficult to determine the absolute concentration of radicals, the comparison of intensities provides only relative information [70].

Although, at least in theory, all mechanochemical methods are suitable for preparing polymers [71,72,73,74], from a practical point of view, some of them are more appropriate for their destruction than preparation [75,76,77], since others are better for mastering or improving the physicochemical properties of a polymer [36,78,79,80,81,82]. In polymer processing, chemical reactions often go unnoticed or only impact the material’s physical properties. Various material destruction applications use mechanochemical processes that generate reactive species (e.g., radicals) [27,83,84].

Long-chain plastics or biopolymers can expand noticeably in elongational flow fields. Forces along the backbone near the center lead to the splitting of chains. New chemical transformations exploit this unique property at site-specific bond cleavage-engineered structural elements, creating mechanophores embedded in the backbone. These mechanophores can have different chemical reaction centers, and their specific combinations have paved the way for so-called “smart” polymers. Various experimental techniques can generate elongated flow fields that allow for studying the mechanochemistry of polymers in solution, as summarized in a review by May and Moore [17].

### 3.1. Cavitation

**Figure 3:**
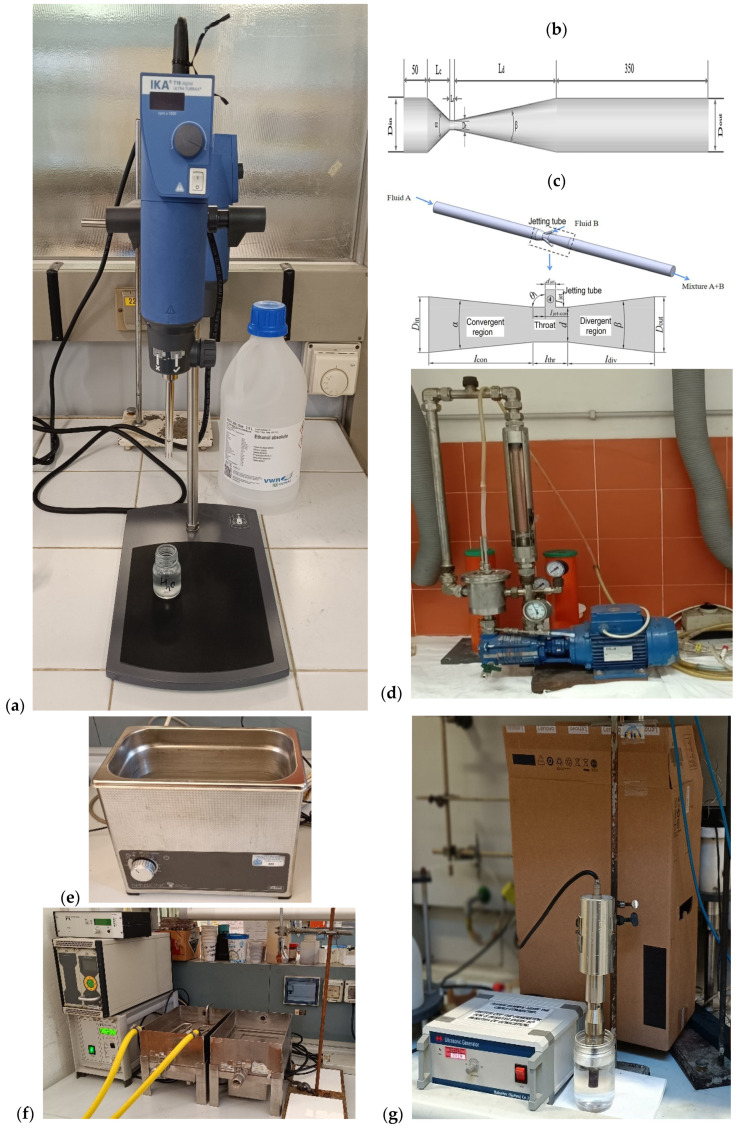
Instruments for cavitation experiments. Pictures of (**a**) and (**d**–**g**) were photographed by the authors. (**a**) Ultra-turrax; (**b**) schematic view of simple Venturi tube, Liu and Li [85]; (**c**) schematic view of Venturi tube with reagent inlet, Tang et al. [86]; (**d**) laboratory-scale Venturi-instrument; (**e**) laboratory (cleaning) bath ultrasonicator with single frequency and energy level; (**f**) laboratory bath sonicator with controllable frequency and energy; (**d**) horn-type laboratory sonicators with controllable energy level.

Some examples of cavitation-based common laboratory instruments are in Figure 3.

#### 3.1.1. Hydrodynamic Cavitation

Cavitation in flowing fluids is a common phenomenon. The physics is simple according to Bernoulli’s law, although the mathematical description of a realistic system is complicated. Various textbooks thoroughly discuss the theoretical and practical aspects [87]. The fundamental theorem states that in an ideal flowing fluid, the following simplified equation (Bernoulli’s equation) holds:

v^2^/2 + g × h + p/ρ = const, (3)

where v is the fluid flow velocity at a point, g is the gravitational acceleration, h is the height above a reference plane, p is the pressure along the streamline, and ρ is the density of the fluid. It means that a change in the fluid flow velocity results in a pressure change in the fluid. Cavitation occurs when rapid pressure changes in a liquid cause numerous small cavities to form, which then implode. Hydrodynamic cavitation uses Venturi tubing or high-speed rotation, in which rapid changes in the flow velocity trigger cavitation. Although this phenomenon usually causes severe damage to rapidly rotating objects, the Venturi tube allows a safer and controllable mechanochemical process. From a practical point of view, the occurrence of cavitation is independent of the event that triggers it.

#### 3.1.2. Ultrasonic-Triggered Cavitation (Sonocavitation)

Typical areas of sonication include the efficient mixing or dissolution and activation of solids, and more recently, its use has extended to macromolecular, micro-, and nanostructures, some biological or medical applications, ultrasound-responsive materials, and crystal design. Traditional mechanochemical protocols require minimal or no solvent, while ultrasound-induced mechanical and chemical effects need a liquid medium. Although ultrasound-induced cavitation is principally identical to hydrodynamic cavitation, its origin is quite different. The cavitation phenomenon is always the result of a rapid drop in pressure in a flowing or swirling liquid. Sonocavitation occurs when the propagation of sonic waves creates high- and low-pressure zones. The nonlinear forces that generate tensile stresses are similar to solid mechanochemistry and are equivalent to cavitation as the high amplitude waves propagate in the liquid phase [88].

The growing demand for natural products has put the spotlight on the need for greener and cleaner technologies, and ultrasound-assisted extraction is an efficient and rapid way to isolate raw materials from a variety of biological sources. However, the industrial application of ultrasound is still limited, and the technology transfer from laboratory to production faces challenges at several stages of industrialization, from process optimization to scale-up, safety, and regulatory issues [28]. Many parameters that influence the physical and chemical changes induced by ultrasound are still not fully understood, and the various kinetic models can provide incorrect predictions for macromolecular degradation [89,90]. Gogate and Prajapat reported that in addition to conventional reaction parameters, such as the concentration, presence of functional groups, and more, the technology-specific operating frequency and power dissipation can significantly influence the reaction mechanism and the behavior of components in the reaction mixture [26]. These effects encompass polymerization–depolymerization processes, the production of nanoparticles or colloidal solutions, or food preparation techniques. In many instances, ultrasonic physical fragmentation often determines chemical transformations.

Meanwhile, although many low-frequency sonication methods are known as low-frequency ultrasonication (10–60 kHz), the frequencies are often in the human hearing range [91]. Appropriate variation in the sonication parameters can change different physical, chemical, and biological effects, resulting in improved target processes. However, the ultrasound-provided beneficial effects on bioprocesses are usually case-specific and rarely public. 

Another classification of sonication uses the energy transferred by the sound as follows:

− Medical imaging and chemical analysis utilize low-power, high-frequency ultrasound in the so-called extended or diagnostic ultrasound frequency range, typically ranging from 2 to 10 MHz.
− High power and low frequencies, i.e., in the range of 20–1000 kHz, are suitable for cleaning, welding, and sonochemical reactions.

High intensity at low frequencies can also destroy cell walls, so these conditions are suitable to kill bacteria [92].

Several ultrasound parameters, such as frequency, intensity, duration, and the like, determine the potentially harmful effects on biomolecules, which may result from the extent and severity of possible cell disruption by heating and free radicals that can attack cellular material during sonication. In a comprehensive review, Rokhina summarized [91] the current state of the art in areas where sonochemistry could be successfully combined with biotechnology to enhance the efficiency of bioprocesses, including biofuel production, bioprocess monitoring, enzyme biocatalysts, biosensors, and biosludge treatment. Table 2 briefly summarizes the sonication parameters of the most common applications.

Ultrasonic baths as laboratory accessories have become widespread in recent decades. While they are principally used for comminution or accelerated dissolution, these simple instruments can also be suitable for chemical transformations. It is often difficult to distinguish between chemical changes governed by ultrasound and the higher reaction rate on an enlarged solid surface or dissolution [97].

The interaction of sound with matter through cavitation occurs on time scales unavailable from other sources. In liquids, cavitation is typically caused not by applied mechanical pressure but by intense sound waves. The alternating compression and expansion regions produced by these waves result in the formation of bubbles with a diameter of up to 100 microns. The bubbles implode in less than a microsecond, heating their contents to nearly 5500 °C [46,98].

Cavitation can produce reactive species from the medium that are not directly related to the energy transfer to the processed material. Instead, these species interact with the chemically reactive domains of the otherwise inert molecule, causing chemical transformation. In terms of the chemical reactions inside the bubble, there are around a hundred reactions involving N_2_, O_2_, H_2_O, OH, H, O, HO_2_, H_2_O_2_, O_3_, N, HNO_x_, NO_x_, and N_2_O [99]. The most typical reactions that generate various radicals are in Table 3.

An appropriate selection of the reaction medium, where the oxygen, or in general, the solubility of gases is lower than in water, or the application of a noble gas atmosphere can eliminate the formation of unwanted reactive species. Single-bubble numerical simulation have shown that at low ultrasound frequencies of 20 kHz and 100 kHz, a peak in the bubble temperature is formed as a function of the acoustic amplitude, i.e., the ultrasound pressure amplitude. The acoustic amplitude is relatively large in typical sonochemical experiments, mostly more than 1 atm. Numerical simulations have also disclosed that there is an optimal bubble temperature for the OH radicals and H_2_O_2_ production. The formation of nitrous oxides continuously consumes the oxidants in the bubbles, resulting in the gradual dissolution of species, such as HNO_x_ and NO_x_, in the bulk water. The reactive components can transform the dissolved molecules. Above 10 W of high-frequency ultrasonic irradiation, H_2_O_2_ formation increases dramatically, and the pH of the aqueous medium decreases in parallel. The H_2_O_2_ content, interestingly, shows a smooth decrease with repeated sonication after a specific relaxation time [109]. At higher ultrasonic frequencies, the vapor fraction at the end of collapse is small because the bubble has less time to expand due to the shorter acoustic period. The bubble temperature increases with the increasing acoustic amplitude and becomes saturated at relatively high acoustic amplitudes. At 300 kHz, the linear resonance radius of a bubble is about 11 μm [110,111]. However, the temperatures reached during single-bubble cavitation in liquids of significant vapor pressure are remarkably limited by the endothermic chemical reactions of the polyatomic compounds in the collapsing bubble [112]. In the real world, the situation is more complicated. The surrounding bubbles and the collision- and implosion-generated secondary acoustic waves strongly influence the bubble pulsations in a multi-bubble system [113,114].

### 3.2. Grinding

**Figure 4:**
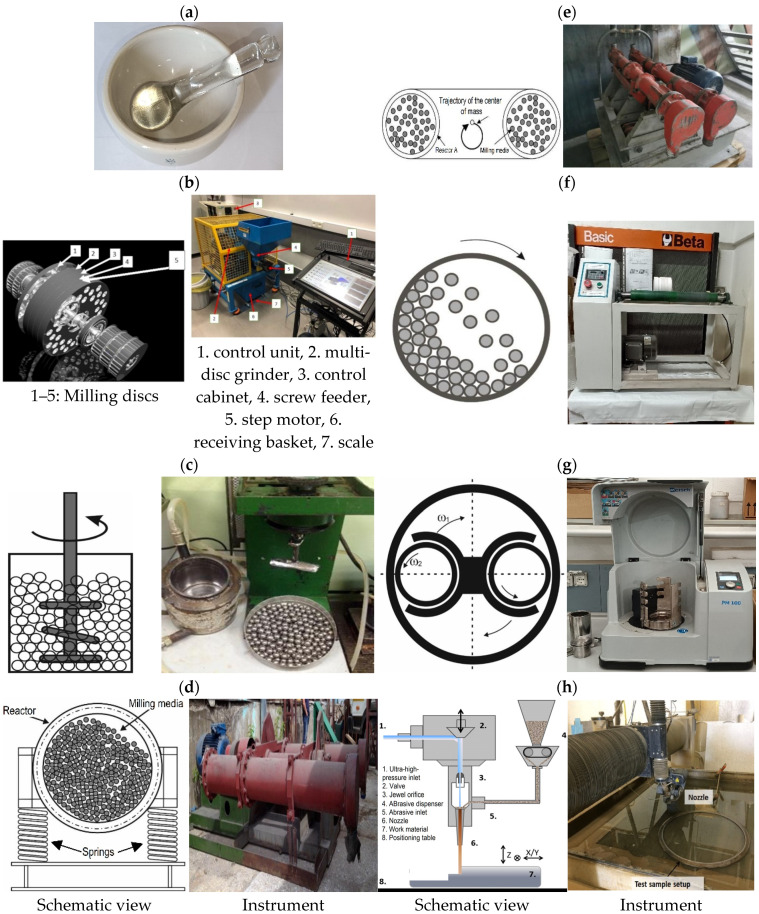
Typical grinding instruments. (**a**) Mortar and pestle; (**b**) disc mill with five milling discs, from Kruszelnicka et al. [115]; (**c**) mixing mill; (**d**) vibrating mill; (**e**) vibrocentrifugal, mill; (**f**) roll mill; (**g**) planetary ball mill; (**h**) abrasive jet mill. Instruments (**a**) and (**f**,**g**) were photographed by the authors; schematic pictures (**c**–**g**) are from Lomovskiy et al. [116]; instruments (**c**–**e**) are from Lomovskiy et al. [116]; schematic and instrument pictures (**h**) are from Holmberg et al. [117].

Grinding uses kinetic energy transfer to produce chemical changes at the atomic or molecular level. Figure 4 shows typical laboratory- or pilot-plant-scale instruments.

Mortars [47] and disc mills [118] are the oldest grinding instruments. While mortars were the first in mechanochemical synthesis, disk mills have been a tool for physical comminution technology for thousands of years. Ball mills are relatively new instruments among comminution techniques and can produce significantly smaller particles than the traditional methods. The advent of more efficient grinding methods has emerged with the demand for their use in various synthetic processes. Developments in material sciences have enabled the design of more energy-efficient mills. Improved energy efficiency has been the driving force behind the development of powder technologies and an increase in the number of synthetic applications. The modeling of physical and chemical processes in ball mills, after the simplifications of the early days, has been accompanied by the advent of modern computers, which have developed simulations of the physical and chemical changes that occur during grinding [39,119,120,121,122]. 

Ling and Baker reported the first mechanochemical experiment of the industrial era, describing the halogenation of quinones in a mortar [40]. While a mortar can be a tool in many organic reaction experiments, its restricted applicability and lack of reproducibility make it a mere curiosity. This technique is typically not a routine method in preparative chemistry, and its utilization aims to pulverize or homogenize small samples.

Disc mills are one of the oldest types of grinding instruments. Their principle of operation is friction, where material passes between two rotating discs, causing particles to grind against the discs. The most common uses of disc mills are grinding grains such as wheat, maize, and other crops, as well as fibrous materials, although roll mills are also frequently used. The process typically produces a grain size range of 300 to 200 µm [116].

Grinding and mechanical pre-treatment effectively improve the reactivity of polymers and vegetable raw materials. Even today, trial and error in equipment selection and the determination of operating parameters is common, and inappropriate equipment selection or operating conditions can result in inefficient grinding, excessive energy consumption, and significant equipment wear. A recent review compares the available grinding techniques and can help in making the right choice to increase the effective surface area, reduce the crystalline region, and disorder the supramolecular structure [116].

The simulation of the motion, collision process, energy transfer, and change in the temperature of small balls during the ball-milling process is complicated, and the actual models for the mechanical, physical, and chemical events in mills fundamentally use two approximations [123]. The kinetic approach uses a simplified description, assuming that the balls behave as particles that do not interfere with each other in motion and no relative slip exists between them. Based on these assumptions, the motion model aims to describe and analyze the forces present in a mill [120,121,124]. An alternative approach is the energy transfer model that concentrates on energy transfer efficiency modes and the influence of the milling-ball energy on the overall outcomes of ball milling. The energy transfer model considers the relationship between the radius, mass, volume, and the grinding medium energy. It hypothesizes that energy transfer occurs through simultaneous collisions and grinding in the ball-milling process, assuming only inelastic collisions where the kinetic energy of the grinding medium is transferred entirely to the matter particles [119,121]. Chattopadhyay et al. highlighted the importance of the instrument’s geometry [120], and Schilz demonstrated that the impact energy of material particles is only related to the rotation ratio, revolution radius, and angular velocity [124]. Many grinding simulations uncovered incongruities between calculated and observed trajectories [125,126], suggesting that a limited number of ball collisions have a favorable impact as friction and shear dominate these collisions. In addition, Maurice and Courtney demonstrated in dynamic simulations that only 4–7% of all crashes have the potential for significant energy conversion [127]. Several energy transfer models assume that ball collisions follow the Hertz impact theory, i.e., the crash process involves wholly elastic collisions, resulting in no energy loss, and the Hertz radius, which represents the radius of the contact area, is significantly smaller than the radius of the impact milling ball. Consequently, the contact surface of the impact is a plane [127]. Product formation is closely tied to the operating conditions of a ball mill. A common approach to optimizing the grinding conditions, reducing friction, and preventing the excessive thermal decomposition of organic molecules and polymers due to the heat generated is through trial and error. However, the kinetic equations used in mills usually overlook frictional events. The formulated kinematic equations depend on the mill design parameters, and the energy transfer is a function of experimental factors such as the grinding frequency, time, number of balls, and size of the balls [119,121]. Recently, simulations have revealed the chaotic behavior of ball-milling systems [128]. 

Burmeister and Kwade analyzed the energy relationships from an engineering perspective. The specific energy, Ei, can be calculated from the number of stress events (SN), the average stress energy (SE), and the total mass of soil material (m_p_), as shown in Equation (3). The stress energy SE_j_ describes the maximum amount of energy (equal to kinetic energy) in interval j that the product particles can receive during an impact event from the relative impact velocity v_j_ and the masses of the colliding bodies m_1_ and m_2_. (SE_j_ = 0.5 × v_j_^2^ × m_1_ × m_2_/(m_1_ + m_2_)) [129,130].

E_i_ = SN × SE/m_p_ (4)

In mechanochemical processes, the thermal effect of the process is crucial [131,132,133], especially when thermosensitive materials are the subject of mechanochemical treatments. Unlike cavitation, where a very-high-temperature effect is in the microsecond range but the bulk temperature practically does not change significantly, the heat load takes longer in ball milling. The weak heat conductivity of solids in the system causes long-lasting thermal stress, but the bulk temperature seldom surpasses 100 °C. A review by Sacher et al. discusses potential methods for reducing the magnitude of this temperature rise [134]. The impact energy can be calculated by the following equation, assuming the impact energy UE is transferred to the material in a single collision process:

UE = C_p_ × ΔT × Q (5)

where Q is the mass of the colliding materials, C_p_ is the weighted average specific heat capacity of materials, and ΔT is the temperature change [123]. In nucleophilic exothermic reactions, the changes in the temperature of the milled materials (and balls + container) showed a saturation curve [135,136,137,138]. At the beginning of the reaction, the temperature of the medium rises quickly but slows down and converges to a more or less stable value. When the exothermic reaction is over, the temperature decreases slowly to another constant value [137]. Usually, the difference between the temperatures of milled solids with and without chemical reaction do not significantly differ. The water content of the ground material can enormously increase the temperature of the milling and can result in some caramelization in the case of carbohydrate substrates [135,136]. In wetting materials, usually 1–2 percent or less of a chemically inert liquid, in planetary ball-mill experiments, the temperature of the grinding media and the ground solids do not differ significantly, regardless of the mass of the ground materials [138]. Takacs and McHenry found similar phenomena in various mills in alloying processes [139].

Typical ball mills include the following fundamental devices: rotary ball mills, planetary ball mills, vibrating ball mills, mixer mills, pin mills, and attritors, often referred to as stirring ball mills, and, of course, the many variants of the listed devices. The exhaustive summary of the main operating characteristics, grinding parameters, advantages and disadvantages, and most common applications are in the reviews of Baláž et al. [140] and Lomovskiy et al. [116]. The conversion of mechanical energy induces reactions between the reagents on the colliding ball surfaces. The contact time is short, in the range of microseconds (10^−4^–10^−3^ s), and the surface temperature can reach 10,000 K, but the bulk temperature is significantly lower and rarely exceeds 100 °C in organic reactions. The bulk temperature of the interior of the grinding jar depends on the state of the reactants and the grinding media since the appearance of the liquid phase fundamentally affects the heat transfer from the reaction site to the bulk material, including the grinding media.

Mechanochemical degradation usually involves the splitting of a macromolecule. Empirical calculations of stress strength in amide- and ester-type polymers have shown that most of the resulting frequency shifts should be negative, primarily due to the anharmonic nature of the atomic potential, resulting in a decreasing force constant with increasing valence coordinate deformation [141,142]. Quantum chemical tools are suitable for calculating the stress required to separate two chemical parts linked by covalent bonds. Unfortunately, in the case of large molecules, calculation using efficient molecular mechanics and low-level (e.g., semiempirical) quantum chemistry is unsuitable for this purpose. Crist et al. pointed out that molecular mechanics and semiempirical techniques lead to erroneous assumptions, and the relative amount of deformation of covalent bonds, bond angles, and internal rotation angles under tension must be inversely proportional to the relative stiffness of the deformation modes. An ab initio method with large basis sets is necessary for calculating the total molecular energy vs. stretching potential energy function. The different molecular deformation modes, i.e., bond stretching, bond bending, and internal rotation, are excited simultaneously and not sequentially at different stress levels [143,144].

Studies of ball-mill process parameters and polymer properties on the mechanochemical degradation of amorphous polymers have shown that the grinding frequency has the highest effect on the degradation rates. Molecular weight and molecular weight dispersity also influence the degradation rates but in an indirect manner similar to ultrasonication treatment. The applicability of the relationships found for polymethacrylates to biomolecules with similar structural characteristics, e.g., ester-linked biomolecules, is unclear [145].

Mechanical processing treatments can not only promote the physical changes in proteins, but the transferred energy can also disrupt weak interactions, such as hydrogen bonds, hydrophobic interactions, and the like, that significantly alter the biochemical functionalities. Although modification of the secondary, tertiary, and quaternary protein structures alone can alter their basic functionalities, these changes are usually reversible, especially in living systems. However, the radicals generated also interact with the covalently bound systems, causing fundamental and irreversible changes in the primary structures. These changes not only affect the processed food components but can also have noticeable effects on living organisms [146].

### 3.3. Extrusion

Extrusion is a process used to produce objects of a fixed cross-sectional profile by forcing material through a die of a desired cross-section. Extrusion has two main advantages over other manufacturing processes, as it is suitable for producing very complex cross-sections and processing brittle materials because the shaping uses only compressive and shear stresses. The high-quality surface of the extrudate allows a high degree of design freedom [147]. Table 4 summarizes the properties and utilization of the most common extrusion processes.

**Figure 5:**
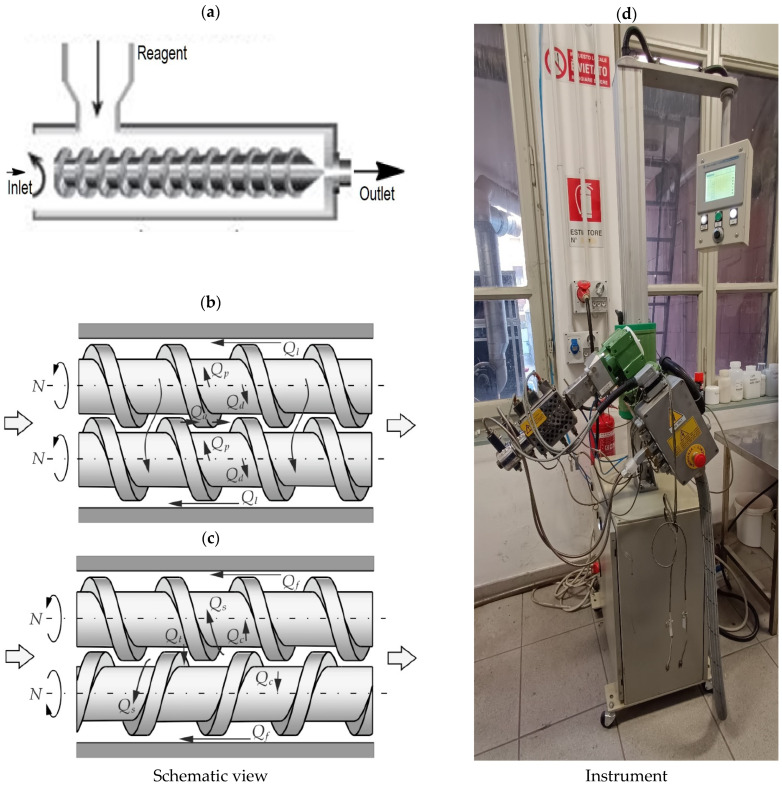
Typical laboratory-scale extruder. (**a**) Single-screw extruder; (**b**) conrotating twin-screw extruder; (**c**) counter-rotating twin-screw extruder; (**d**) laboratory single-screw extruder. Schematic picture (**a**) is from Calcio Gaudino et al. [156]; (**b**,**c**) is from Lewandowski et al. [157]; (**d**) was photographed by the authors in the authors’ laboratory, producer: GIMAC International S.r.l. (Castronno, Italy).

Extrusion has many variations, such as continuous or semi-continuous processes and dry or wet technologies, and it can use hot or cold materials. Commonly extruded materials include metals, polymers, ceramics, concrete, modeling clay, and food products. Figure 5 demonstrates the working mode of typical laboratory extrusion machines.

Drawing is a similar process that uses tensile strength to pull the product through the die. Its limitation relies on its one-step process, making it suitable only for simpler shapes. Drawing is the primary method of producing wire and tubing.

Although many publications promise to discuss the degradation of biopolymers, most of them stick to generalities such as hydrolysis, chain shortening, radical formation, transesterification, etc., and focus only on the effect of different process parameters on some inline and offline properties of the product, such as compressibility, rheology, color, etc. [158,159,160,161]. Near-IR spectroscopy is an appropriate tool for monitoring most degradation processes associated with water, hydrolysis, or transesterification. Inline near-IR monitoring of the behavior of polyhydroxyalkanoates in a twin-screw extruder has shown that the C-H and C=O overtone bands can be suitable for monitoring the chemical changes during the process. The mathematical manipulation of the raw spectral data and principal component analysis can provide comprehensive and direct feedback on the overall reaction rate compared to offline methods such as ^1^H-NMR, gel permeation chromatography, or differential scanning calorimetry. Offline product monitoring faces several challenges, and X. Liu et al. pointed out that analytical methods and parameters that, at first glance, seem secondary may cause chemical changes in the product. The incorrectly conducted analytical experiments of the components can ultimately lead to false results in the analysis of the finalized product [162]. The problems raised in their paper can also have dramatic implications for many previously published results or conclusions. The applicability of various analytical techniques is summarized, and the interrelation between the independent variables, extrusion conditions, material properties, and structure in extrusion processing are discussed [163,164]. In a detailed study of extruder parameters, for example, the screw profile and speed, temperature, residence time, and pre/post-processing operations in reactive extrusion, Guiao found that since the extruder parameters significantly determine the transformation of the materials treated, the product purity is more post-processing-dependent [164].

Thermal degradation during recycling is primarily hydrolytic due to moisture and high temperatures (β-cleavage and transesterification), which can compromise processing and recycling. The use of appropriate stabilizers can also increase the number of recyclable polymers [165]. Most degradation processes involving water, hydrolysis, or transesterification can be monitored inline using near-infrared spectroscopy. Inline near-infrared monitoring of the behavior of polyhydroxyalkanoates in a twin-screw extruder has demonstrated that the C-H and C=O overtone bands can also be used to monitor chemical changes in the various segments of the instrument used. The mathematical manipulation of the raw spectral data and principal component analysis can provide comprehensive and direct feedback on the overall reaction rate compared to offline methods such as 1H-NMR, gel permeation chromatography, or differential scanning calorimetry. This allows for parameter variations to be linked not only to the immediate chemical transformations but also to changes in the physicochemical properties of the final product. The use of artificial intelligence can also help to understand the deeper interdependencies in the processes [166].

Reactive extrusion processes combine the difficulties of mastering classical polymer processes with the problems of controlling the chemical reaction under extreme conditions, e.g., high-viscosity medium, high temperature, short residence time, and more. It can be hard to capture all the process aspects, including complex interactions between parameters, which are essential for proper industrial technology design and optimization. Numerical modeling can be a powerful tool to overcome these difficulties, although the combination of flow simulation in complex geometries with reaction kinetics and rheological behavior is always challenging [167]. In addition, the required information is rarely publicly available. Reactive extrusion is not a new process, but it has evolved rapidly since the early 2000s and is now increasingly common in the chemical modification of polymers. The extruder works as a continuous chemical reactor, and besides the classical functions of a screw extruder (solid conveying, melting, mixing, pumping), additional functions, such as control of the reactions, are necessary. Compared to classical batch processes in solution, reactive extrusion can use a molten state, i.e., without solvents, an extruder can work with very viscous products, and the operating conditions are much more flexible. As long as the various additives fundamentally alter the thermal and mechanical properties of existing plastics or biopolymers, the typical purpose of reactions with small molecules in reactive extrusion is to functionalize polymers or, generally, to derivatize macromolecules. Various radical initiators can also be appropriate to modify macromolecules by grafting small molecules into an existing structure or by creating or breaking cross-links in the polymer. High and ultrahigh temperatures are suitable for thermochemical recycling and degradation processes. Modeling approaches for reactive extrusion processes generally use conventional chemical engineering methods based on single or coupled reactors [168]. It is worth noting, though, that several disadvantages make the overall picture more nuanced. The residence time in the extruder is only a few minutes, which allows for only fast reactions in the process. Moreover, for example, the restricted cooling capacity of the equipment poses heavy challenges in managing highly exothermic reactions [167].

The precise formulation of the kinetic equations that describe the reactions remains unclear because the various kinetic constants involved are often not independent [169,170]. Even minor inaccuracies in such constants can lead to significant discrepancies between the simulations and experiments. To address this issue, rheokinetic models have attempted to describe the changes in viscosity and other material parameters based on the extent of the reaction. However, only mixed success has been reported in the implementations [78,167,171]. The eventual non-Newtonian behavior of the reaction mixture presents additional challenges in both the technology and formulation as well as in the solution of the kinetic equations [172].

## 4. Mechanochemical Transformation of Biopolymers

In plastics, the C-C, C-H, or O-H bond-breaking energy in organic molecules and plastics is in the 85–110 kcal/mol range [13,173], but in biomolecules, significantly less energy, 75–85 kcal/mol or even less, appears [174,175]. In plastics, the dissociation energy of the C-N bond is close to 30 kcal/mol [176,177], but in peptides, with a few exceptions, it is predominantly in the range of 5–20 kcal/mol [178].

Akbulatov and Boulatov’s systematic critical review discusses the limitations of different approaches to quantify and validate mechanochemical reactivity [173]. Although their focus was on sonicated polymer solutions to identify the unresolved issues in making polymer mechanochemistry a rigorous, quantitative field, their recommended points for future work touch on all types of mechanochemistry. With a few exceptions, the deep mechanistic and quantitative understanding of mechanochemical phenomena is still uncertain. They also suggested focusing on solving the following most fundamental current problems in polymer mechanochemistry:

− Identifying new reactivity patterns is necessary due to the inadequacy of current experimental tools that are insufficient for studying force-inhibited reactions in mechanochemistry.
− The development of efficient and accurate methods to determine the activation free energies of mechanistically distinct reactions as a function of applied force, to understand deeply the effect of mechanical stress on the chemical reactivity, and to enable the efficient design of materials with the desired mechanochemical profile.
− The development of quantitative microscopic models of mechanochemistry in sonicated solutions, as only a few publications have reported reactions in sonicated polymer solutions and solids under stress. To date, sonication experiments do not appear to add much to simple qualitative considerations based on molecular geometry in rationalizing the mechanochemical behavior of solids or in selecting monomers to achieve the desired solid-state mechanochemical reaction.
− In fact, the effect of polymer architecture on mechanochemical properties is unknown. The chain dynamics of topologically complex polymers often differ significantly from those of linear analogs.
− New mechanochromic compounds possess a force/velocity profile suitable for precise quantitative analysis, both computationally and experimentally, via single-molecule force spectroscopy. These compounds offer a vast range of customizations through straightforward chemical modifications. Further, their reaction to mechanical stress is reversible and long-lasting and induced by the energy input.
− Laying the fundamentals of accurate models of polymer mechanochemistry, rather than relying on localized reactions, by incorporating macroscopic control parameters, such as stress tensors of solid materials, the formulation of pressure gradients in flow systems, the effect of acoustic power fluxes, and the like. Moreover, the estimation of single-chain forces by bulk rates and product distributions of reactions with well-established microscopic mechanochemical kinetics and mechanisms as a function of macroscopic control parameters.
− The description of mechanochemical reaction rates as a function of excitation time and the quantitation of the accumulation of single-chain forces by local deformation of strained materials. The experiments need accurate activation and standard free energies for the reactions as a function of local reactive and single-chain stress and reliable tools for quantifying reaction rates in solids. The empirical data available so far suggest that mechanochromism offers a potentially convenient method for quantifying reaction progress in loaded solids by studying changes in optical properties.
− A comprehensive analysis of the nature, extent, mechanisms, and macroscopic manifestations of mechanochemical phenomena in valuable technological processes is necessary. Although empirical evidence shows the increasing importance of mechanochemistry in polymers, there remains an unexplored need for a thorough review [173].

**Figure 6:**
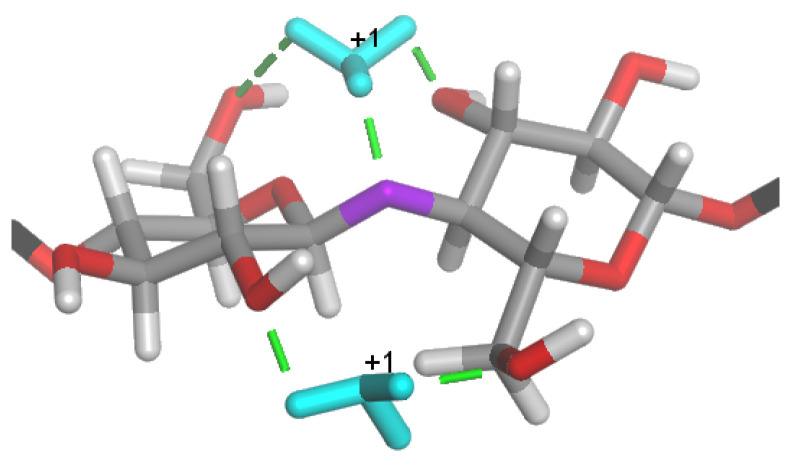
Protonation of cellobiose subunit in aqueous acidic solution according to Loerbroks [181] (with the permission of Chem. Eur. J, Wiley) and Schüth [179] (with the permission of Catal. Today, Elsevier). Cyan represents protonated water, glucosidic oxygen is violet, and green dashes show H-bonds.

Cellulose is one of the most abundant polysaccharides in nature, so its degradation studies under mechanochemical treatments are at the forefront of research. The β(1→4) glucosidic linkage of cellulose is chemically more stable than the α(1→4) and α(1→6) types in amylose and starch. Quantum chemical studies using density functional theory on cellobiose showed that in an aqueous acidic solution, glycosidic oxygen protonation accounts for 90% of the value of the apparent activation energy for hydrolysis. The higher proton affinity of water compared to glucosidic oxygen inhibits the proton transfer from H3O^+^ to the glycosidic oxygen in β-glycosides due to the proximity of the more basic O(2), O(3′), O(5), and O(6′), as seen in Figure 6. The less flexible β-glycosidic linkage compared to the α-linkage makes the cellulosic polysaccharide structures rigid, and the crystalline domains give efficient protection against hydrolysis. The conformational change in the free cellobiose moiety is close to 3.0 kcal/mol, about 10% of the total activation energy, and is significantly less than that of the polysaccharide chain [179,180].

Oppositely to previously published statements, W.-C. Liu demonstrated that highly branched starch, amylopectin, may experience fragmentation under high temperatures in a twin-screw extruder despite the α(1->6) glucosidic linkage being traditionally considered more stable than the α(1->4) one [182]. The molecular weight distribution of extruded low- and high-amylopectin corn starches showed opposite behavior under different extrusion conditions. However, drawing general conclusions on the effect of the physical, thermal, and pretreatment history of starches from diverse sources during extrusion on product properties is currently unclear. Li et al. found a moderate correlation between the extrusion of starches with a high amylopectin content and the intensity of physical stress. The amylopectin content in starches with a high amylose content decreased significantly, almost independently of the applied shear force intensities. On the other hand, physical stress caused minimal changes in the debranched starches [183].

Biopolymers, like plastics, can be easily modified with organic molecules containing reactive or readily transformable functional groups. For example, epoxides offer an attractive modification potential for polymer backbones, but their reactivity under mechanochemical agitation can sometimes be low [184]. Epoxides can eventually also be formed internally from various carbohydrate or polyhydroxalkane moieties [185,186], or the biopolymers can react with externally added epoxides [136,187,188,189,190]. Although the oxirane groups are reactive and hydrolysis occurs almost always parallelly with the addition reaction, the change in the configuration of the secondary carbon can lead to significant conformational changes in the polymer chain. However, epoxides are potentially harmful to living organisms, mainly because of their ability to modify genetic material, so reducing the residual reagent content in the product is a priority.

The much less hazardous carboxylic acids and esters are biologically much safer, but the physiological effects of residual reagents, due to their reduced reactivity, also need consideration. These safer chemical residues can also affect the product’s bioavailability or the food’s taste.

Mechanical forces, sonication, or rapid flow of polymer solutions can transform macromolecular chains or their segments into elongated, non-equilibrium geometries. However, the vast amount of data collected has only slowly allowed us to break out of the phenomenological era of polymer mechanochemistry. Over the past decade, not only have there been technological changes or explosive advances in analytical methods, but the raw biomaterials involved in the processes have also often undergone significant changes. 

Biopolymers exhibit distinctive behavior compared to petroleum-based plastics due to their sensitivity to shear and thermal stress and have the potential for chemical structure degradation during extrusion. In such instances, high shear and temperature may trigger unwanted chemical reactions despite the short residence times, while upholding the components’ integrity and molecular weight is desirable. Although traditional reactive extrusion yields satisfactory outcomes, the modeling and simulations can further optimize the system’s performance. The use of artificial intelligence can fundamentally improve the understanding of processes of extrusion [191,192].

Generally speaking, mechanochemical transformations affect the transformation of biopolymers through a series of hydrolytic, dehydration–rehydration, elimination, and addition reactions. In most, but not all, the reactions that generate radical or acidic species initiate the chemical transformations. The presence of radical-generating agents, inorganic or organic acids, air, or water can accelerate or control the chemical reactions. Meanwhile, various inhibitors can make the polymers more resistant to the physical or thermal stresses induced during processing.

### 4.1. Cavitation

Ultrasonic waves in liquids generate chemical and mechanical impacts that significantly contribute to chemical reactions. Water-soluble polymers typically exhibit frequency-independent properties [26]. While external chemicals are not necessary to initiate radical reactions, sonication can still cause desired and undesired chemical changes in both artificial and biological polymers due to the radicals from dissolved gases. Although, for the time being, technological advances have established ultrasonic tools for use on an industrial scale, the combination of sonication with thermal, mechanical, photochemical stimuli, or flow chemistry is still in the research phase [27].

**Figure 7:**
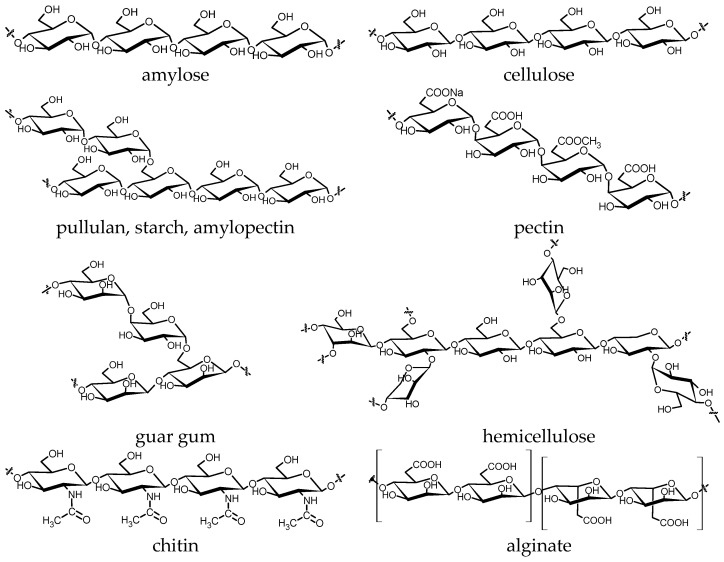
Most typical structures in common (1→4) and (1→6) linked polysaccharides.

Natural polymers, such as carbohydrates with various glycosidic bonds—starches and amyloses, celluloses and hemicelluloses, pectins, chitins—or lignins are part of everyday life. Most common carbohydrate polymers have dominantly (1→4) and some (1→2) or (1→6) glycosidic bonds and comprise cellulose, amylose, starch, amylopectin, pectin, and pullulan, as seen in Figure 7. Many living species produce these polysaccharides, and even mineral oils and coals are of an organic origin from the historical time of the earth. Slowly depleting mineral resources have drawn attention to the acceleration of “mineralization” processes, and scientists everywhere are making extensive efforts to replace vanishing mineral raw materials. Mechanochemical techniques, especially ultrasound-induced cavitation bubble collapse, are energy-efficient and green technologies for gaining value-added materials from various biomasses [75,193,194].

Under identical experimental conditions, the limiting molar mass below which degradation does not occur is five times lower for amylose than cellulose. The degradation rate of amylose was almost three times higher (≈2.7 × 10^−6^ min^−1^) than that of cellulose (≈0.9 × 10^−6^ min^−1^) upon 47 kHz sonication at 185 W. Despite the invariance in the main polymeric structural features, the average molar mass distributions, polydispersities, and analyte volumes showed extensive changes during degradation. The persistence length appears to be a fundamental parameter embodying the minimum continuous path length and flexibility requirements of degradation. These results modify the path theory of transient elongation flow degradation by isolating the degradation rate in (1→4)-linked polysaccharides [193,194].

After brief agitation with Ultra-turrax, α-chitin showed an increased porosity and surface area due to deacylation. The cavitation treatment resulted in a significant increase in the ionic dye uptake and a reduction in the saturation time compared to the raw material [195]. Some studies on glucose have reported that ESR showed the appearance of similar radicals in γ-irradiation and ultrasonication [196]. The cooperation between sonication and radical attack plays a role in ester degradation in polymers. Abstraction of a hydrogen atom significantly alters the potential energy surface of the bond scission, reducing the force needed to break the bond by approximately half, from nearly 4.7 nN to around 2.5 nN [197]. Ultrasonic treatment of aqueous chitin suspensions showed similar effects to gamma irradiation [198,199]. The reduced molecular weight improved the lipid absorption of modified chitin, which may act as an active ingredient in dietary food additives and can be a functional tool in lipid intake reduction. The deacylation process and molecular weight reduction are associated with the OH-radical-induced chain scission of chitosan [200]. Ultrasound or λ-ray irradiation dramatically increased charged moieties in α-D-glucoside oligomers of nearly 4000 Da with and without oxygen in the system [201].

Sonolysis, γ-radiolysis, and acid hydrolysis in aqueous solutions of carboxymethylated cellulose showed that sonochemical degradation occurs both by mechanical rupture of the macromolecular chain and by the action of free radicals generated by cavitation bubbles. The contributions of the two mechanisms showed a strong dependence on the sonication conditions [202].

The natural chemical erosion of bio-elements in water is cavitation-driven due to the production of mineral and organic acids, acidic polysaccharides, and the activation or deactivation of enzymes. Artificial decalcification helps to isolate organic skeletons by removing organic components [203]. 

Striegler raised the possibility that polymers may suffer some degradations, even during simple column chromatography (size exclusion chromatography). A detailed study has demonstrated that high-molecular-weight compact polymers, such as PAMAM dendrimers, are less sensitive to mechanical stress than moderately branched or linear polymers. Although it is hard to localize whether the degradation occurs predominantly in the interstitial medium or the pore boundaries, the publication highlighted that structural changes can occur even under moderately mild conditions [204].

A recent comprehensive analysis by Huang et al. [205] presents the latest overview of the available data on the disruption of intermolecular associations caused by ultrasound, sonosensitizers, microbubbles, and sound-driven micro- and nanorobots. Interactions between high-frequency sound waves and advanced materials produce various biochemical products and enhanced mechanical effects, generating potential biomedical applications such as biosensing, diagnostic imaging, and therapeutic and clinical applications [205].

Low-energy, medium-frequency ultrasonic treatment of a dilute solution of waxy rice starch significantly affected the physicochemical properties of starch granules. Even though the size of the starch particles and the SEM examination of their surface did not show dramatic changes, the viscosity of the treated samples decreased, so the pasting properties near the gelation temperature changed [206]. The ultrasonic degradation of methyl methacrylate polymer by the nitrogen oxide radicals formed indicated the labile C-C bonds in various polymers containing ester functionalities [207].

Comparative studies of hydrodynamic (3000 rpm)- and ultrasonic (25–80 kHz)-induced cavitation of wheat straw powder showed minimal differences in NaOH solution (10% NaOH vs. solids) at different temperatures. In both cases, the carbohydrate composition of the extract and ash was nearly identical but significantly different from that of the untreated wheat straw powder. Similar sonication experiments without NaOH showed about doubled amounts of ash and more than ten percent less extracted carbohydrates [208]. Sonication of *Eucalyptus grandis* in a dilute KOH solution in a moderately warm reaction mixture can increase the weight of extractable hemicelluloses, which offers noticeable potential for industrial-scale hemicellulose production by ultrasonic treatment. However, the sonication slightly decreased the molecular weight of hemicelluloses but increased the xylose content of the extract [209].

**Figure 8:**
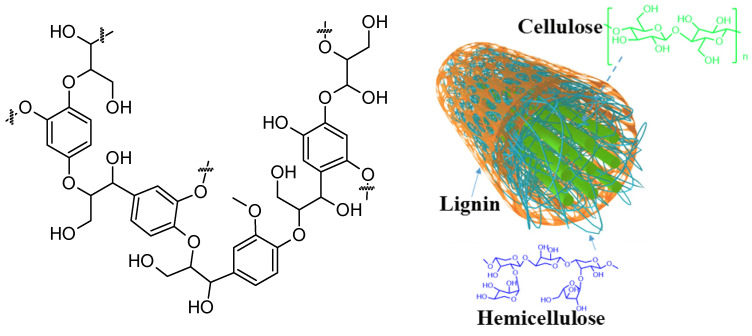
Most typical structure of lignin and lignocellulose. Lignocellulose structure adopted from Zhang et al. [213].

Mechanical forces can induce conformational changes in the pyranose ring that facilitate the cleavage of the glycosidic bond [210]. In lignins, ball milling can also disrupt the crystalline domains of the cellulose, increasing the accessibility of reagents and catalysts to the cellulose fiber. Hydrolysis of the β(1→4) glycosidic linkages relies more on conformational changes than the α-glycosidic linkage [211,212]. Lignin mainly occurs in conjunction with cellulose or hemicellulose in the cell walls of plants, as illustrated in Figure 8.

Acetylated starch is one of the most commonly produced industrial starch derivatives. Mechanochemical methods, such as milling or ultrasonication, are suitable green technologies for acetylation. The acetyl groups on the glucoside units significantly affect the starch functionalities, while the acetylation minimally influences the physicochemical properties of multiscale-ordered starch structures [32].

Galactomannans are linear heterogeneous polysaccharides of mannose and galactose available in plant seeds and microbial sources. The mannose/galactose characterizes these gums and influences their solubility, as a higher galactose content provides increased aqueous solubility. Among the tested mechanochemical methods, ultrasonic and radiation were the most effective in galactomannan depolymerization [33]. The higher number of carbon atoms in the functional groups of the polymer results in a higher depolymerization rate, and in general, a lower polymer concentration improves the depolymerization rate [26,29].

Mechanochemical pretreatment of lignins can improve the microwave valorization process. Both ball-milling and ultrasonic treatment effectively activate the biomass and increase the lignin extraction [75].

Zhurkov et al. studied polyamides and hypothesized that the magnitude and lifetime of the tensile stress correlate with the stiffness of the polymer chain and that the breaking of C-Cα-CONHR depends only on these factors and the temperature [214]. ESR studies of silk protein degradation confirmed that the primary reaction step involves the cleavage of C-Cα to form NH-CαHR and CONH radicals [215]. The rigidity of the polypeptide structure causes an increase in the rate of mechanical degradation of various polymers containing predominantly peptide bonds, such as proteins, polyamino acids, and the like [216,217].

Ultrasonication at 850 MHz frequency inactivated *A. pullulans*, similar to low-frequency sonication, although *A. pullulans*, due to its specific cell wall properties, is generally more resistant to sonication than other yeast-like fungi and bacteria [109].

PCR tests use DNA amplification to detect viral DNA in various samples. Blood samples require heparin to prevent blood clotting, but the heparin molecule inhibits DNA amplification. Degradation and fragmentation of the macromolecule can reduce the inhibitory effect. Sonication greatly accelerates the depolymerization process above 65 °C in the presence of nitrous acid, hydrogen peroxide, and ascorbic acid. The application of ultrasound reduced the multiplication threshold of canine parvovirus at low pH by 15–25 times and improved the test sensitivity [218].

Sonopharmacology combines controlled drug release with cavitation-induced cleavage of covalent bonds, such as a disulfide bond for a drug attached to a linear polymer. Sonication of the composite at 20 kHz effectively releases thiol from the microgels [219,220,221]. The non-covalent version of sonopharmacology uses polymeric micelles and ultrasound disintegration of the microcapsule for controlled and targeted drug delivery [72].

Oxidative folding is essential for forming a bioactive three-dimensional structure in polypeptide chains with cysteine residues. In living organisms, selenium-containing proteins also play a crucial role in many redox processes, as has recently been discovered. Since a folded state with the right disulfide (S-S) combination is essential, the redox properties of selenoproteins are equally vital for an organism. Selective cleavage of Se-Se bonds is difficult with classical reactions because of their similarity to S-S bonds. Sonication of diselenide-bonded polymers can cleave the Se-Se bond, regenerating selenoproteins [222,223,224,225].

High-frequency ultrasound, at 850 MHz, successfully disinfects wastewater. Inactivation occurs through the membrane disruption by cavitation, and chemical destruction of the genetic components by the free radicals principally generates superoxide and peroxide radicals. The inactivation of the *E. aerogenes*, *S. epidermis*, *B. subtilis*, *A. pullulans*, and yeast was successful at all growth stages, while *A. pullulans* was the most resistant to ultrasonic treatment [226].

The treatment of several bacteria, *E. aerogenes*, *B. subtilis*, and *S. epidermidis*, at high frequencies resulted in ultrasound-power-independent inactivation at both the exponential and stationary phases of the bacteria. The bacterial cells with capsules were more resistant to the ultrasound treatment. The bacteria were more resistant to low-frequency sonication, probably due to the lower concentration of the hydroxyl and hydroperoxide radicals generated [109]. The inactivation of the bacteria was related to the thickness of cell walls and the generated radicals that directly attacked the bacterial cells by reacting with the peroxidase enzyme released from the cell, as cavitation causes mechanical damage [227,228]. Free radicals within cells also cause damage to nucleic acids, proteins, liposomes, and membranes [229].

### 4.2. Grinding

The transformation of biopolymers into soluble products is one of the most challenging problems in the cellulose industry. Catalytic amounts of strong acids, such as sulfuric or hydrochloric acid, and the impregnation of cellulosic substrates brought many advantages to solid-state reactions. The hydrolysis became highly effective, as grinding the acid-impregnated cellulose quantitatively converted the polysaccharides into water-soluble oligosaccharides within two hours. By comparison, an aqueous acidic solution at 130 °C converts the formed soluble glucan fraction into glucose and 96% of the xylans into xylose within an hour with a 91% conversion rate. Ball milling of other natural sources, like wood, sugarcane bagasse, or switchgrass, also convert the insoluble solids into water-soluble products, such as oligosaccharides and lignin fragments [230].

ESR spectroscopy confirmed the formation of radicals during the ball milling of air-dried cellulose. The degradation rate increased in the presence of iodine because iodine can prevent the recombination of some of the free radicals generated during grinding. On the other hand, in the grinding of dried and moderately wet (air-dried) cellulose, the iodine consumption of the samples remained unchanged, making it likely that heterolytic cleavage of the glucosidic units was negligible. However, in the presence of toluene, the ball milling of cellulose benzyl- and cyclohexyl iodide was also detected. These side reactions were probably the result of a reaction between cellulose radicals and solvent molecules or hypoiodite formed due to iodine absorption by alkoxy radicals [231].

The cellulose degradation under mechanochemical conditions is a radical chain reaction, as confirmed by ESR spectra of the reaction and IR spectroscopy of low-molecular-weight reaction products [232,233].

Although the idea of ball-milling starch into polyacrylamide polymers was first mooted in the middle of the last century [51,234,235], mixing a natural polymer, such as cellulose, starch, alginate, or carrageenan, with plastics is still more a scientific curiosity that is only slowly finding its way into everyday practice [187,228,236,237]. The high shear stress of extrusion is more commonly utilized industrially than grinding for the combination of natural and hydrocarbon-based polymers [238,239]. Although both technologies are suitable for the copolymerization of plastics and natural polymers, the degradation process of biopolymers is currently outside the focus of research because radical polymerization and degradation products are difficult to distinguish in very complicated mixed structures.

Bleached eucalyptus cellulose treated with dilute oxalic acid is suitable for cellulose nanofiber production in a disc mill. A process model utilized a reaction kinetics-based combination of hydrolysis factors to provide a quantitative description of xylan dissolution and depolymerization in eucalyptus cellulose. The model successfully predicted the degree of polymerization of disc-milled fibrils using the combined hydrolysis factor, grinding time, or milling energy consumption. The applied cellulose longitudinal hierarchical model confirmed that weak acid hydrolysis is suitable for cellulose nanofibril production with significantly reduced energy input without negatively affecting the mechanical strength of the fibrils [240,241,242,243]. Mechanical fibrillation successfully produced highly carboxymethylated cellulose transparent films [244].

Both ball and disc mills are also suitable for the gram-scale radical oxidation of benzyl hydroxyl groups in lignins by the selective cleavage of aryl-Cα in the presence of (2,2,6,6-tetramethylpiperidin-1-yl)-oxyl, oxon, and KBr [245]. Sodium hydroxide is suitable for depolymerizing organosolv lignin in a mixer ball mill. A rapid depolymerization occurs within minutes, but extending the grinding time to 8 h results in a slower decrease in average molecular weight. The progress of depolymerization appears to be significantly influenced by re-polymerization reactions between the products. Scavengers, such as methanol, can suppress re-polymerization, and a similar effect can also be achieved by adjusting the moisture content of the starting material. These modifications result in lower average molecular weights and higher monomer yields [246].

Although a vibrating ball mill significantly altered the starch structure of wheat endosperm by reducing the A-type crystalline fraction, the chemical degradation was less than ten percent. The amylopectin fraction behaved oppositely, and longitudinal-vibration-induced damage significantly reduced the viscosity and hydrolytic resistance of amylopectin [247].

Succinic anhydride can esterify kraft lignin in a zirconia jar in a planetary ball mill. Vulcanization of carboxyl-terminated butadiene-acrylonitrile-terminated liquid rubber with a combination of epoxy resin and lignin ester has significantly improved the fracture toughness and impact resistance of rubber, albeit reducing the flexural strength [35].

Ball milling can accelerate the transesterification reaction of methyl oleate and lignin, and the product is suitable for blending polylactic acid through melt extrusion [35]. Plant-cell-wall lignocellulose is composed of cellulose (≈45%), hemicellulose (≈25%), and lignin (<35%). It has extensive hydrogen bonds that hold the molecule in a crystalline structure, making it inaccessible to hydrolytic enzymes [248]. Ball milling combined with ultrasonic treatment of cellulosic and hemicellulosic raw materials with classical alkaline methods increased the saccharification rates [209].

Approximately 30% of non-fossil organic carbon is lignin, so the conversion of this biopolymer is of paramount importance for the efficient use of industrial feedstocks and wastes [249]. Ball milling allows for the temporary metal- and solvent-free cleavage of β-O-4 bonds in various lignins, and the moisture content of the raw materials does not substantially affect the reaction efficiency. As well as being a green process, it is more efficient than conventional synthesis, which uses catalytic amounts of base and dimethyl carbonate to produce 2-aryloxyvinylbenzene derivatives [250,251].

Polymeric carbohydrates with reducing ends are suitable for chemical conversion to raw materials for fuel and renewable hydrogen production. The mechanocatalytic pretreatment of lignocellulosic barley straw allows for efficient saccharification, but the process also requires potassium pyrosulfate for a more effective transformation [252].

A one-pot green approach based on the combination of solid components in a vibrating ball mill can significantly improve the efficiency of fermentation of glucose derivatives to bioethanol through the high sugar content, and the precipitation of lignins provides sulfur-free products [179,253,254,255].

The SuperMassColloider is a device that maximizes the mechanochemical-frictional-forces-induced transformations. A short but intense grinding process produces a small, uniformly distributed spherical powder that also efficiently initiates chemical reactions such as the oxalic acid hydrolysis or depolymerization of different types of cellulose. The reported technology is appropriate for the economic and green production of various bio-based fine chemicals [242].

Solid-state reactions often produce oligosaccharides that are more water-soluble than crude celluloses. This is because the smaller molecules generally have a higher reactivity than cellulose and hemicellulose themselves and are better feedstocks for a more efficient production of 5-hydroxymethylfurfural and furfural [256].

### 4.3. Extrusion

Extrusion, steam explosion, or ultrasonic treatment of soluble dietary fiber from buckwheat bran significantly have been shown to affect the fiber content and monosaccharide composition. The physical stresses essentially changed the surface of the bran to a porous and wrinkled texture, reduced the crystallinity, and improved the thermal stability, rheological and hydration properties, and enzymolytic properties [257,258].

The two-step extrusion process has produced polypropylene/TiO_2_/nano-cellulose hybrids with a high tensile strength and a higher Young’s modulus. The physical-stress-induced structural changes in the cellulose and polypropylene superstructures resulted in good nucleation properties and higher crystallization temperatures [259].

Chaetoglobosin A is an alkaloid compound with anticancer properties and is produced by fungi. Extrusion of lignocellulose in the presence of glycerol and aqueous NaHCO_3_ solution resulted in accelerated degradation and also reduced the particle size and crystallinity of the components but effectively increased the yield of chaetoglobosin A isolation from rice straw by *C. globosum* CGMCC 6882 [260].

Ball milling of lignin and methyl oleate resulted in oleic acid transesterification. Melt extrusion of the product and polylactic acid resulted in a composite with higher tensile properties and reduced degradability due to the lower number of free phenolic residues [35].

Hot melt extrusion and compression molding stabilized plasticized pullulan. Kinetic studies suggest that microphase separation leads to a disorientation phenomenon during swelling [261].

Various plant materials can serve as raw materials for food production, e.g., meat analogs, with unique physical and chemical characteristics [262,263]. In the hot extrusion process, the shearing forces and temperature significantly impact the taste and aroma of the product. However, the proportion of reducing sugars and protein has the most substantial influence on the final flavor composition [264]. The Maillard reaction occurs in the hot phase, and the flavor components, which determine the product palatability, are impacted by the extrusion process parameters, as reported in numerous publications [265,266,267,268].

Thermoplastic starch properties, as prepared by mechanochemical plasticization, differ from traditional plastics and cannot be compared to them. Micro- or nano-cellulose addition is also necessary to improve the polymer biodegradability, moldability, and elasticity during the melt or internal mixing extrusion of thermoplastic starch, as reported in numerous publications and summarized by Cataño et al. [81].

The copolymerization of corn starch with derivatized maleic anhydride in a twin-screw extruder modified the rheological and thermoplastic properties of starch and significantly improved the shear resistance. Repeated grinding and extrusion of the copolymers changed the extensional viscosity and reduced the unmelted granule content [269].

The extrusion of silicate–polyamide hybrid systems in a twin-screw extruder can improve rheological properties due to an extended structural network over the polymer matrix due to polymer–silicate interactions. Nanoscale silicate exfoliation with macroscopic preferential orientation can significantly affect the protein’s secondary and tertiary structures during the extrusion [270]. High-intensity ultrasound and extrusion together yield ionic condensation reactions and degradation. Although the simultaneous processes have been shown to increase the polyamide’s molecular weight and crystallinity via mechanochemical and sonochemical effects, they have also resulted in a slim deterioration of the mechanical properties. Nevertheless, such combined processes on biomolecules are still partially studied [271,272]. The bio-assimilation of common polymers with plasticized starches by extrusion did not significantly change the melting properties of the plastics but fundamentally improved their degradability in simulated soil despite the increased crystallinity and morphological changes [34].

Cast extrusion of 3-glycidoxypropyltrimethoxysilane-derivatized starch is a fundamental component in biodegradable plastic films, where the interfacial adhesion between the plastic and the extruded starch modified the elongation-at-break percentage [273].

An alternative extrusion method to hot extrusion cooking in food technology uses continuous heating. This technique is high-moisture extrusion, which has more complex elementary steps due to mixing classical and mechanochemical reactions. Studying and identifying the principal technological parameters are even more complicated in the mixed reaction mechanism [31,274,275].

Low-moisture extrusion significantly altered the structural and functional properties of biopolymers in adlay millet, reducing the content of dominant components in the following order: starch > protein > fiber > lipids. The degraded starch exhibited poorer hot water swelling properties, and the extrusion-induced Maillard reaction and caramelization, in addition to reducing the degree of gelatinization, darkened the color of the adlay flour [276].

Continuous extrusion of spun polyethylene glycol acrylate and sodium alginate gel solutions or calcium alginate fiber significantly reduced the apparent viscosity of an alginate solution by nearly 93%. The ionic cross-linking of the macromolecules by the dynamic shaping process of solidification noticeably influenced the stretching and flexibility in the biomolecule. The higher polyethylene glycol acrylate content significantly reduced the storage and loss modulus [277].

An extruded starch blended with glycerin and urea showed a lower tensile strength, but at the same time, the elongation-at-break percentage increased. The probable explanation is that glycerin acts as a plasticizer, while urea weakens the interactions of the starch molecules. The degree of shear stress significantly impacts the production of composite crystallinity in either a static or dynamic mixer-coupled single-screw extruder [278]. The extrusion reduced the molecular weight of the starch, and its biodegradability was slightly higher than that of native starch [279]. Extrusion of corn starch with water, glycerol, and stearic acid showed a gelatinization temperature dependent on the glycerol content. The lipids formed complexes with amylose during extrusion, and although the starch fragmented even under highly annealed conditions, its degradation was minimal [280]. The viscosity–temperature curves also demonstrated that the inclusion of polyvinyl alcohol into the starch resulted in an increased shear rate and apex at the optimal starch level. Furthermore, the extrusion process with polyvinyl alcohol exhibited a faster degradation of color components compared to carbohydrates or polyol [281].

Polyhydroxyalkanoates are bio-based microbial polyesters of many bacteria that are used as intracellular storage carbon and energy sources against starvation. Their stiffness, elasticity, crystallinity, and degradability are tunable by the monomer composition and process parameters during production and post-synthetic processing. The degradation products, either from monomeric or oligomeric sources, typically do not have any harmful impact on living cells, nor on animal or human tissues. These compounds hold clinical significance across various medical fields, including surgery, therapeutics, and tissue engineering. Traditional processing techniques for the manufacture of polyhydroxyalkanoate medical devices, such as melt-spinning, melt extrusion, or solvent evaporation, and emerging processing techniques, such as 3D printing, computer-aided wet-spinning, laser perforation, and electrospinning, are summarized in recent reviews by Koller and Mukherjee [37,282].

Structural changes in combined thermal and physical forces make alginates suitable bio-ink hydrogels for 3D-extrusion-printed biomedical 3D scaffolds or tissue-like structures with hierarchical vasculature [82].

Thermal degradation of sugars and amino acids and depolymerization of macromolecules by high-temperature, short-time extrusion modify the physicochemical and functional properties of the raw materials. This technology holds promise for the expanded use of non-conventional ingredients as food/feed due to its industrially feasible, highly productive, and efficient method and its enhanced ability to retain thermally degradable nutrients during cooking. The current knowledge of the high-temperature, short-time extrusion cooking process does not allow us to draw general conclusions. For example, different extrusion temperatures may alter starch digestibility and digestion characteristics, but the starch digestion kinetics do not appear to change, but the starch hydrolysis index is significantly modified [30].

The food industry widely uses acetylated starch as a food additive. However, limited information is available on the effects of acetylation on starch structure and functionalities, even in the case of advanced acetylation technologies. Although both the sonication and extrusion processes are suitable for acetylation, the effects of sonication or extrusion are still unclear, i.e., the differences between the mechanochemical- and acetylation-induced changes in the starch structure are currently unknown. In most cases, the production parameters of both technologies are also unknown or at least not published [32].

Starch, lignin, and hemicelluloses are suitable raw materials for biopolymer-based controlled drug delivery hydrogels by reactive extrusion. The process reduces the molecular weight of all three macromolecules, and a higher plasticizer content can increase the weight loss rate of the hydrogels. Catalytic amounts of acids can reduce the degradation rate and significantly affect the compressive modulus of the hydrogels produced [283].

The reactive extrusion process eliminates the discontinuity of the low-yield batch reaction, the major weakness of starch cationization, and provides improved strength, retention of fillers and fines, and dewatering of paper pulps. At the end of the process, the positively charged starch component remains broken during the repulping process [167]. The production of plant-based meat analogs widely uses the high-moisture extrusion technique, but despite extensive studies, the scientific basis of the process is still unclear. A recent review summarizes the hypotheses behind the physics of the functional tasks and principal structural changes in high-moisture extrusion [31]. Density functional quantum chemical calculations have correctly estimated the material bulk properties and were in good agreement with the experimental results [284].

Polylactic acid reacted with chitosan amine moieties during the reactive extrusion process. The copolymer showed better self-stabilizing properties and a higher resistance to physical stress than PLA with an emulsifier [285]. Reactive grafting of silk nanocrystals onto PLA macromolecules improved the rheological and thermal properties, as confirmed by ^1^H-NMR and Fourier transform infrared studies. The higher degree of grafting and cross-linking restricts the movement of silk fibroin chain segments by controlling the crystallization. The reduced degradation grade improves the PLA recyclability and rheological properties over branched and cross-linked PLA in all reprocessing cycles [286].

Spent polyesters as a lignin matrix can be a sustainable resource for producing high-performance thermoplastics from industrial waste and by-products. The solvent-free melt-blending process efficiently regulates lignin dispersion and interfacial interactions, resulting in more mechanically durable lignin in the grafted recycled polyethylene terephthalate. Thermal treatment of molten substances can prevent the breakdown of lignin and the loss of volatile compounds. Long-chain fatty acid oils can enhance the processability of polyethylene terephthalate at moderate temperatures without causing the charring of lignin. Heat treatment increases the total phenolic hydroxyl content by reducing the aliphatic hydroxyl content and cleaving β-O-4 ether linkages. The results of the structural transformation lignin are a higher molar mass, better thermal stability, and slightly smaller dispersed lignin domains. Furthermore, grafting lignin into the blended matrix via reactive extrusion can improve product formability [287].

## 5. Conclusions and Perspectives

Modeling the mechanical-stress-generated molecular changes in small model compounds is widely possible in cavitation and grinding processes. Although the thermal effects in the cavitation methods are the highest, the actual knowledge of the reaction kinetics can handle it. Many publications on extrusion often refer to the possible chemical changes in general terms and focus on the physicochemical differences caused by changes in various parameters, neglecting a detailed discussion of the chemical changes in the molecules. The model reactions in extrusion processes can show a higher divergence, as a generalized theory on the extrusion chemical processes is still missing. Many extrusion parameters are instrument- and process-optimized, and the model reactions cannot accurately predict the process results. Additionally, the typical extrusions, except in the alloy industry, work with complex materials, and the purity and molecular composition show a wide variety, particularly in the food industry.

Physical stresses resulting from cavitation or collision phenomena can modify the primary, secondary, and tertiary structures of biopolymers. Alterations in the secondary or tertiary interactions, such as hydrogen bonding or hydrophobic/hydrophilic interactions leading to different spatial arrangements, are typically reversible under mechanical forces. Conversely, the generating or breaking of S-S or Se-Se bonds, primarily in proteins, results in irreversible modifications. The stability of a glycosidic bond in polysaccharides is sensitive for both the hydroxyl group positions involved in the linkage, namely 1→2, 1→3, 1→4, or 1→6, as well as the bond configuration, α or β. The most stable and least flexible bond is β(1→4) in polymeric carbohydrates. Mechanical stress can potentially induce functional group migration and polymerization or depolymerization. Mechanochemical reactions can generate radicals from various sources, such as air dissolved in liquids or homo- or heterolytic bond scission in the polymeric chain.

Fragmentation probability tends to decrease significantly below a limited chain length in polysaccharides, much like in plastics. Fragmented high-molecular-weight biopolymers typically do not lead to principally different chemical or physicochemical characteristics of biopolymers. Physical shock modification of natural polymers can yield a sustainable production of fine chemicals and fuels from biological feedstocks, modified degradation properties of plastics, and even ground or wastewater decontamination.

A systematic critical review by Akbulatov and Boulatov [173] outlined the principal topics of future interest in mechanochemistry. Intensive experimental works are still necessary to provide a well-founded scientific description and understanding of the events in cavitation and grinding processes despite the numerous efforts in fundamental theoretical approximations and simulation methods. Since the physics and chemistry of extrusion processes are intricate, fewer scientific considerations and more empirical factors shape the optimization of the various extrusion parameters. Future studies must also focus on understanding the degradation of biopolymers resulting from the copolymerization of plastics and polymers of natural origin. It is necessary to integrate advanced artificial-intelligence-based models to predict the degradability of biopolymers and study the process in line and in real time to improve their physical and chemical properties.

## Figures and Tables

**Table 1:** Approximate bond dissociation energies in kJ/mol that are relevant for many biopolymers.

Bond	Aliphatic	Non-Aliphatic	Ref.
N-H	314		[14]
O-H	428		[14]
S-H	344	314	[15]
Se-H	305		[14]
C-C	284–368	410	[16]
C=C	615		[16]
C-H	381–410	427–435	[16]
C-O	350–389	381–410	[16]
C=O	749		[14]
CO-O	395–414	368–381	[15]
C-N	293–343	460	[16]
C=N	615		[16]
O-O	498		[14]
C-S	699		[14]
S-S	429		[14]
C-Se	582		[14]
Se-Se	333		[14]

**Table 2:** Low-frequency ultrasound application in biotechnology (extracted from [91] with the permission of Trends Biotechnol, Elsevier, if otherwise not stated).

Transformation	Frequency [kHz]	Power [W]	Sonication Time [min]
Transesterification for biofuel production	20–48	120–1200	10–140
Vegetable oil emulsification for biofuel production	40	700–1200	120
Peroxidase-catalyzed degradation of phenol	423	5.5	20–60
Protease-catalyzed oxidation of untanned leather waste	40	N/A	120
Cellulase-catalyzed degradation of distillery wastewater	22.5	120	30–120
Immunosensor B. subtilis var. niger	1900–3000	N/A	3 ^a^
Laccase-catalyzed decolorization	150–850	42–120	60–540
Tyrosinase-laccase immobilization	20–40	600	>10 ^b^
Increase in dehydrogenate activity of waste-activated sludge	35	80	10
Anaerobic digestion of waste sludge	20–40	600	60 ^c^
Draining waste sludge	20	300–1000	15–480 ^d^

^(a)^ [93]; ^(b)^ [94]; ^(c)^ [95]; ^(d)^ [96].

**Table 3:** Typical radical formation reaction during the ultrasonication of an aqueous solution.

Reaction Type	Reaction	Reference
Dissociation of molecules	O_2_ → 2O• N_2_ → 2N• N_2_O + O• → 2NO•	[99,100,101] [99]
Hydroxy radical formation	H_2_O → •H + •OH •H + O_2_ → •OH + O	[102]
Peroxide and peroxy radical formation	O + H_2_O → H_2_O_2_ •H + O_2_ → HO_2_• O + •OH → •OOH •OH + H_2_O → H_2_O_2_•	[103] [102] [101] [103]
Radical transfer	N_2_ + •OH → N_2_O + H• •O• + N_2_O → 2NO• CO_3_^2−^ + •OH → •CO_3_^−^ + OH− HCO_3_^2−^ + •OH → •CO_3_^−^ + H_2_O	[104] [104] [105] [105]
Formation of the hydrated electron	•H + OH− ⇌ H_2_O + e_aq_-	[100]
Absorption of the hydrated electron	e_aq_- + H_2_O → OH− + H• e_aq_- + H+ → •H e_aq_- + O_2_ → 2O• e_aq_- + N_2_ → 2N•	[100] [100] [106] [106]
Recombination of radicals	•H + •H → H_2_ •H + •OH → H_2_O •OOH + H• → H_2_O_2_	[100,101] [107,108] [103]

**Table 4:** Various extrusion methods and their typical characteristics.

Extrusion Type	Operating Temperature	Typical Subjects	Characteristics and Utilization	Ref.
Hot	350–2000 °C	Metals (alloys), glass, foods (e.g., orange peel)	High machinery and maintenance costs, tearing, blistering of product, and material engineering	[148]
Hot cooking	50–200 °C	Food	Cooking, denaturation, and texturizing macromolecules	[149]
Hot melt	30–150 °C	Organic and inorganic chemicals, plastics, food	Chemical reaction, polymer, pharmaceutical post-processing, and microencapsulation	[150]
Warm	100–1000 °C	Metals, plastics, food	Polymer and food post-processing, alloy production with special purity characteristics	[148,151]
Cold	20–50 °C	Metals, plastics, pharmaceutical ingredients	High pressure, fast extrusion speed, minimal oxidation, high-stability products, rough surface, polymers, and pharmaceutical post-processing	[148,152]
Friction	200–400 °C	Plastics, foods (chips, flakes, etc.)	Energy-efficient, no preheating of feed materials, polymers, and food post-processing	[148,153]
Micro	0–200 °C	Metals, polymers, foods	Uniform small particle distribution (<1 mm), 3D printing, and microelectronic lithography	[148,154,155]

## Data Availability

Not applicable.
